# Performance in emotion recognition and theory of mind tasks in social anxiety and generalized anxiety disorders: a systematic review and meta-analysis

**DOI:** 10.3389/fpsyt.2023.1192683

**Published:** 2023-05-19

**Authors:** Sandra Baez, María Alejandra Tangarife, Gabriela Davila-Mejia, Martha Trujillo-Güiza, Diego A. Forero

**Affiliations:** ^1^Universidad de los Andes, Bogotá, Colombia; ^2^Facultad de Medicina, Universidad Antonio Nariño, Bogotá, Colombia; ^3^School of Health and Sport Sciences, Fundación Universitaria del Área Andina, Bogotá, Colombia

**Keywords:** social anxiety disorder, generalized anxiety disorder, emotion recognition, theory of mind, social cognition

## Abstract

Social cognition impairments may be associated with poor functional outcomes, symptoms, and disability in social anxiety disorder (SAD) and generalized anxiety disorder (GAD). This meta-analysis aims to determine if emotion recognition and theory of mind (ToM) are impaired in SAD or GAD compared to healthy controls. A systematic review was conducted in electronic databases (PubMed, PsycNet, and Web of Science) to retrieve studies assessing emotion recognition and/or ToM in patients with SAD or GAD, compared to healthy controls, up to March 2022. Meta-analyses using random-effects models were conducted. We identified 21 eligible studies: 13 reported emotion recognition and 10 ToM outcomes, with 585 SAD patients, 178 GAD patients, and 753 controls. Compared to controls, patients with SAD exhibited impairments in emotion recognition (SMD = −0.32, CI = −0.47 – −0.16, *z* = −3.97, *p* < 0.0001) and ToM (SMD = −0.44, CI = −0.83 –0.04, *z* = −2.18, *p* < 0.01). Results for GAD were inconclusive due to the limited number of studies meeting the inclusion criteria (two for each domain). Relevant demographic and clinical variables (age, sex, education level, and anxiety scores) were not significantly correlated with emotion recognition or ToM impairments in SAD and GAD. Further studies employing ecological measures with larger and homogenous samples are needed to better delineate the factors influencing social cognition outcomes in both SAD and GAD.

## 1. Introduction

Anxiety disorders are considered ones of the most disabling psychiatric disorders, ranking among the top 25 leading causes of burden of disease worldwide (1). People with anxiety disorders show remarkable functional impairments (2, 3) and decreased quality of life (4, 5). Social cognition impairments are a significant and common feature associated with poor functional outcomes in anxiety disorders (6–9) and are believed to contribute to both symptoms and disability (7, 8). Emotion recognition and theory of mind (ToM) are two social cognition domains critical to successful social and interpersonal functioning. These two domains are especially relevant for anxiety disorders characterized by social impairment, such as generalized anxiety disorder (GAD) and social anxiety disorder (SAD, also known as social phobia).

GAD is a chronic and disabling disorder characterized by excessive, uncontrollable worry, and anticipatory anxiety, which often results in severe cognitive, occupational, and social dysfunction (3, 5). Social anxiety disorder (SAD) is characterized by a persistent, excessive fear, and avoidance of social and performance situations. Both disorders are associated with severe occupational dysfunction and marked social and interpersonal impairments (4, 6). Given the severity and chronicity of social and interpersonal impairments associated with GAD and SAD, it is relevant to understand the underlying social-cognitive mechanisms. However, to date, these mechanisms are not well understood due to the heterogeneity of symptoms in anxiety disorders and the heterogeneity in studies’ methodologies.

Emotion recognition and ToM are two social cognition domains crucial to successful interpersonal interactions, which share conceptual and neuroanatomical overlaps (10), and have suggested to be impaired in GAD and SAD (11–14). However, results regarding these two domains in patients with GAD or SAD are inconclusive and meta-analytic evidence on these social cognition processes is scarce.

Some studies in the literature addressing emotion recognition in GAD or SAD demonstrate impairments (11–14), while others find no significant differences between patients and healthy controls (6, 15–21). The same is true for ToM results, with studies showing lower accuracies in patients than in healthy controls (20, 22–25), no significant differences between groups (6, 26), or even overmentalizing in patients with SAD (27, 28). Besides the uneven results regarding emotion recognition and ToM abilities in patients with GAD or SAD, meta-analytic evidence on these social cognition processes which may underlie interpersonal impairments in these two anxiety disorders is scarce.

Only one meta-analysis (7) has addressed the social cognition performance of adults with anxiety disorders, including both GAD and SAD. The results showed that patients with SAD or GAD exhibited attributional biases, and that other social cognition domains (including emotion recognition and ToM) were not affected. Notably, this meta-analysis included only two studies on emotion recognition in GAD and identified a gap in knowledge for ToM. Thus, there is no recent meta-analytic evidence on performance on emotion recognition and ToM tasks in adults with SAD or GAD, and no meta-analysis has examined ToM abilities in adults with GAD.

Considering these antecedents, the purposes of this study are (1) to sum up and update what is known from the existing literature about emotion recognition and ToM abilities of adults with GAD and SAD, and (2) to determine, through a meta-analysis, whether these abilities are impaired in GAD and SAD adults, compared to healthy controls. We also tested whether emotion recognition and ToM performance in GAD and SAD are associated with relevant variables such as the severity of anxiety, sex, age, and years of formal education.

## 2. Methods

The presentation of this systematic review and meta-analysis was conducted according to the Preferred Reporting Items for Systematic Reviews and Meta-analyses (PRISMA 2020) guidelines (29). We did not register our protocol in online databases. However, review methods such as the databases used, search terms, and inclusion and exclusion criteria, were established *a priori* and there were not deviations from them.

### 2.1. Systematic review

We conducted a systematic literature review to describe and examine the characteristics of studies assessing emotion recognition and ToM in adults with GAD or SAD, compared with healthy controls.

### 2.2. Search and study selection

**Figure 1:**
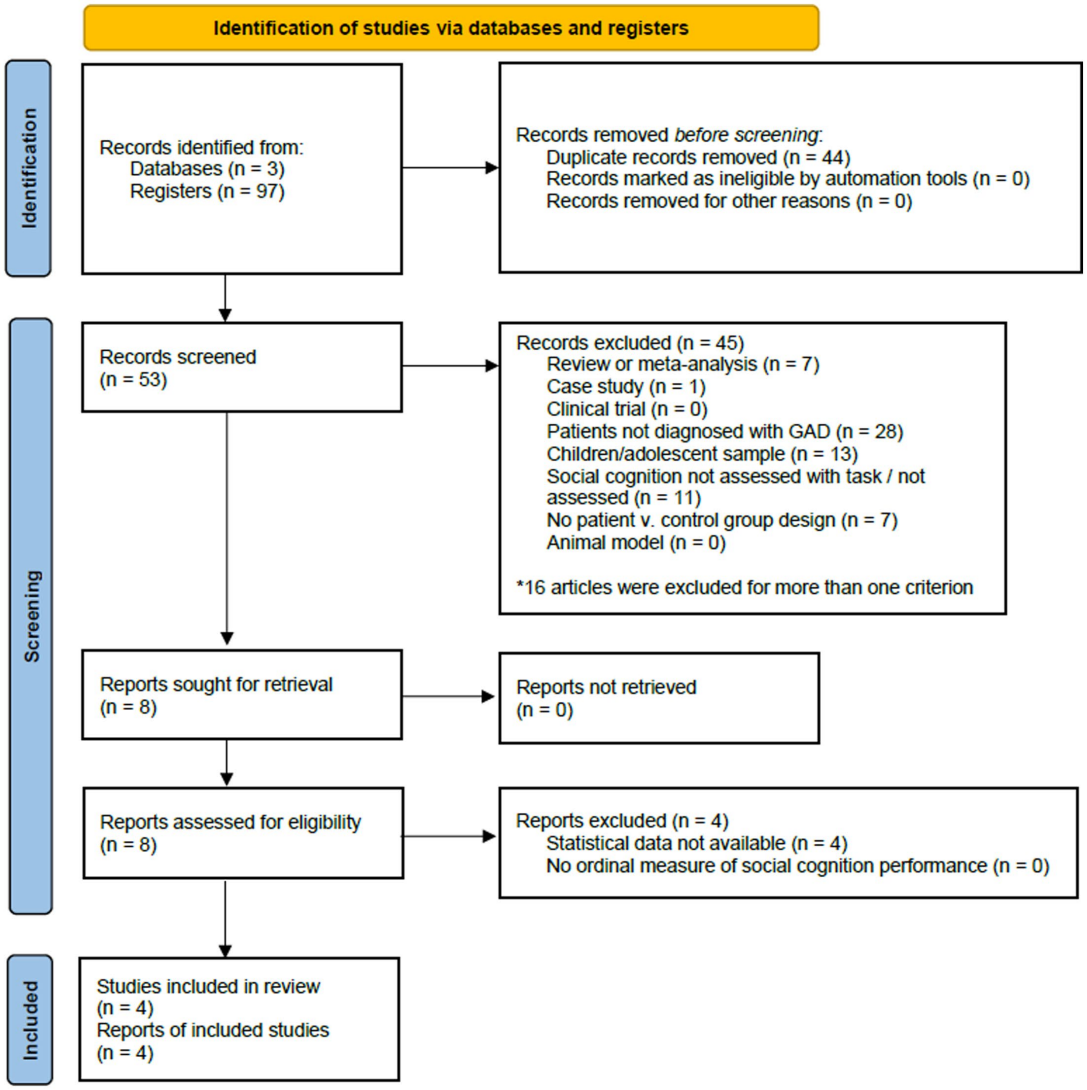
Preferred reporting items for systematic reviews and meta-analyses (PRISMA) flowchart displaying study screening and selection process for studies in generalized anxiety disorder.

**Figure 2:**
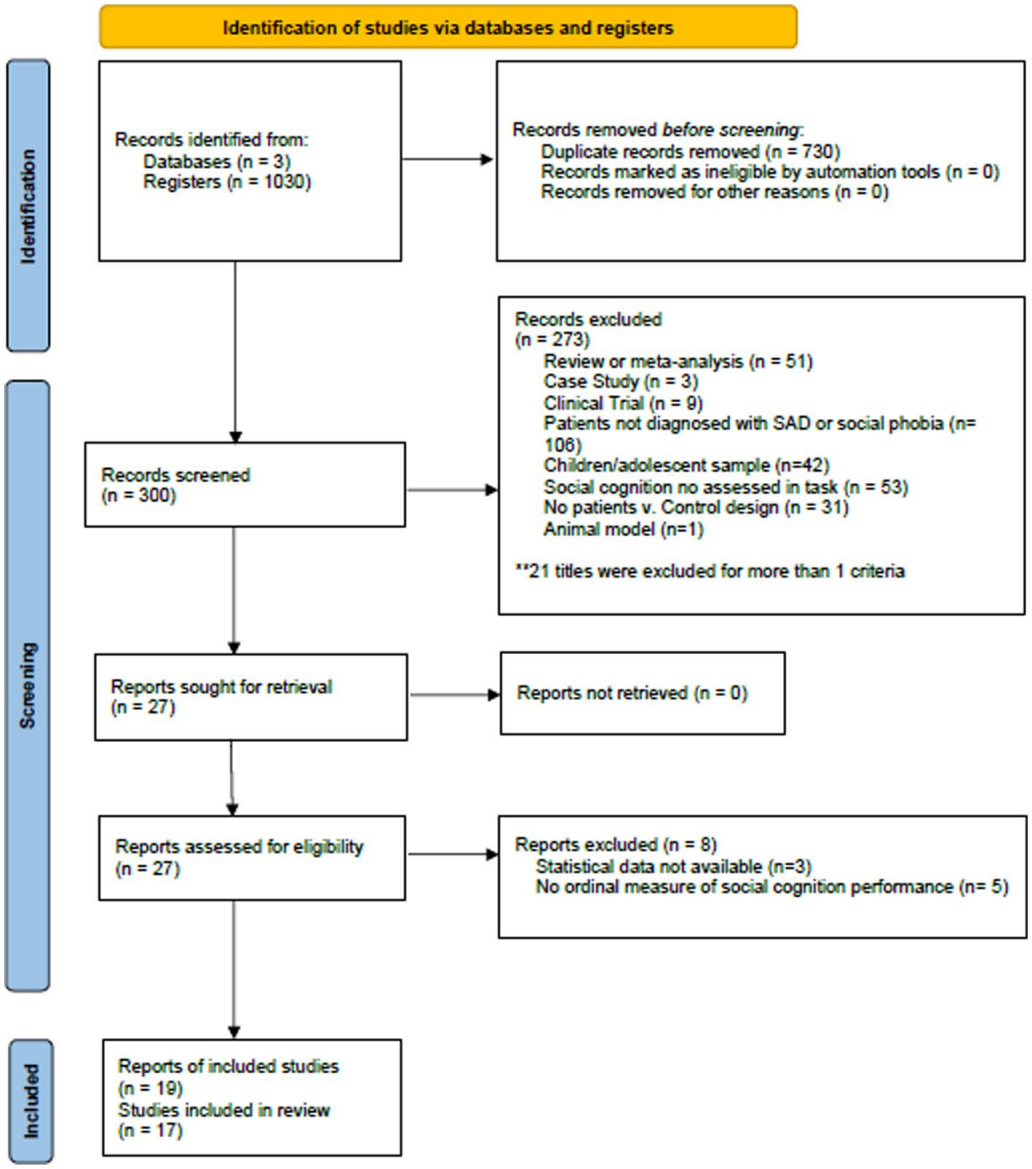
PRISMA flowchart displaying study screening and selection process for studies in social anxiety disorder.

We searched three databases (PubMed, PsycNet, and Web of Science) (30, 31) to identify eligible studies up to February 2022 (last updated on 17 March 2022). The combination of keywords employed for the search and selection process is shown in Figures 1, 2. We conducted two independent searches, one for GAD and another for SAD. Searches were limited to English-language publications and human participants. Titles and abstracts were independently screened by two investigators (MAT and GD), and disagreements were resolved by discussion and requesting a third author’s opinion whenever needed (SB). Full texts of articles were retrieved and read by two authors (MAT and GD) and those meeting all the eligibility criteria were included.

Eligibility criteria for study inclusion were as follows: (1) the study must include a group of patients diagnosed with GAD or SAD, according to the *DSM-IV* or *DSM-V* criteria, (2) the study included a healthy control group without a history of psychiatric or neurological disorders, (3) the samples were adults (above 18 years old), (4) study used data from a performance-based measure of emotion recognition or ToM, (5) article published in English, (6) studies involving human participants, and (7) published in peer-reviewed journals. There was no restriction on the country of origin and the year of publication.

Exclusion criteria were: (1) single case study, (2) non-clinical outcome study (i.e., studies validating social cognition measures), (3) participants without a clinical diagnosis of GAD or SAD, (4) treatments or interventions with no measurement of social cognition at baseline, (5) population-based studies or clinical trials, (6) review, meta-analysis, theoretical, or opinion articles, (7) studies that did not provide adequate data to calculate mean and standard deviations of emotion recognition or ToM tasks (authors were contacted to obtain the data where this was not provided in the manuscript), (8) study without a healthy control group, (9) study reporting questionnaires or self-report measures of social cognition, (10) study with animal models, and (11) study with children or adolescent samples. We excluded studies reporting self-report questionnaires of social cognition because they do not provide emotion recognition or ToM accuracy scores, which constituted our primary outcome measures.

### 2.3. Data extraction

Data of interest were extracted independently by two authors (MAT, GD), and checked by a third one (SB). The following variables were extracted from eligible studies: demographic data of the participants, clinical scales (anxiety and depression measures), and emotion recognition and ToM outcome measures. When available, participant characteristics were also extracted, including the number of participants in each group, age at assessment, sex, and years of formal education. In most cases, data extracted included means and standard deviations or group means of the outcome measures of social cognition. Tables 1, 2 summarize the different emotion recognition and ToM tasks used. The overall accuracy scores in these tests were considered the primary outcome measures. If results at multiple time points were reported, the baseline data were extracted. We contacted the corresponding authors to obtain required data that was not reported. Studies were excluded if required data was not obtained after at least two attempts.

**Table 1:** Characteristics of the studies included for emotion recognition tests.

Author	Emotion recognition test	Principal diagnosis (*n*) and comparison groups (*n*)	Demographic characteristics			Clinical scales		Main results
			GAD/SAD mean age +/− SD Sex (female %)	HC mean age +/− SD Sex (female %)	Years of education mean +/− SD (GAD-SAD) (HC)	Depression scale mean +/− SD (GAD-SAD) (HC)	Anxiety scale mean +/− SD (GAD-SAD) (HC)	
Tetik et al., 2022	ETR	SAD (comorbidity with other psychiatric disorders) = 26 Comparison group: HC = 26	27.68 +/−8.33 31%	27.15 +/− 6.69 23%	SAD = 15.15 +/−2.92 HC =15.83+/− 2.64	Not reported	LSAS: SAD = 68.8+/−20.35 HC = 23.7+/−12.4	Overall scores were significantly different between groups.
Mathai, Rai, & Behere, 2021	Pictorial Emotion Stroop Test	SAD = 27 Comparison group: HC = 26	27.4 +/−8.6 41%	24.4 +/−7.2 62%	SAD = 12.4+/−2.4 HC = 13.4+/−2.5	Not reported	SIAS: SAD =43.9 +/− 13.79 HC =19.9+/−9.68	There were no significant differences between SAD patients and HC in the PEST accuracy.
Bayraktutan et al., 2020	FID	SAD (comorbid with ADHD, MDD) = 36 Comparison group: HC = 30	22.02 +/−0.33 44%	21.2 +/−0.37 43%	SAD = 14.19+/−0.41 HC = 14.23+/−0.41	HAM-D: SAD = 9.86+/−19.76 HC = 18.7+/−11.62	LSAS: SAD = 73.14+/−19.76 HC = 18.7+/−11.62	There were no statistically significant differences between groups.
Oh et al., 2018	FER task	SAD (comorbidity with other psychiatric disorders) = 56 Comparison group: HC = 56	27.25 +/−9.6 46%	25.76+/−5.0 45%	SAD = 14.73+/−2.03 HC = 15.43+/−2.66	Not reported	LSAS: SAD =74.47+/−26.89 HC =21.38+/−17.2	SAD group obtained significantly worst accuracy in the emotion recognition task compared to HC.
Pepper et al., 2018	FEEST, movie still task	SAD = 64 Comparison groups: Autism = 53 Early psychosis = 51 HC = 31	21.75 +/−4.38 47%	24.77 +/−6.08 39%	Not reported	DASS-21: SAD =24.38 +/−11.22 HC = 4.71+/−5.36	Not reported	There were no significant differences in any of the tests between SAD and HC.
Tseng et al., 2017	DANVA-2-TW	SAD (comorbidity with MDD, PTDS, OCD) = 31 Comparison group: HC = 31	29.58 +/−10.36 45%	30.9 +/−9.33 45%	SAD = 14.94 ± 2.32 HC = 15.95 ± 2.79	HAM-D: Not reported	LSAS: SAD = 79.52 ± 29.06 Not reported in HC	Overall scores were not significantly different between groups. However, SAD participants exhibited significantly lower accuracy in recognizing facial and prosodic emotions of fear, compared to HC.
Maoz et al., 2016	Face Emotion Recognition task	SAD = 37 Comparison group: HC = 21	28.4 +/−6.8 38%	25.5 +/−5.5 57%	Not reported	Not reported	LSAS: SAD = 82.2+/−12.4 HC = 17.9+/−7.7	There were no significant differences between groups in overall accuracy. However, SAD participants judged a higher proportion of faces as angry, compared to HC.
Fonzo et al., 2015	Emotion Face Assessment Task	GAD = 21 Comparison group: HC = 12	33.93 +/− 10.55 76%	30 +/−10.21 58%	GAD = 15.87 +/− 2.20 HC = 16+/−1.96	Not Reported	STAI: GAD = 53.20+/−8.47 HC = 29.90 +/−5.04	There were no significant differences between GAD and HC in the average task accuracy.
Fonzno et al., 2014	Emotion Recognition Task	GAD = 15 Comparison group: HC = 15 Panic Disorder = 15	29.68 +/− 9.55 80%	27.58 +/−3.00 60%	GAD = 15.76 +/− 2.09 HC =15.08 +/− 0.55	QIDS: GAD = 8.76+/− 4.27 HC = 1.83 +/− 0.98	PSWQ: GAD = 17.90 +/− 2.36 HC = 12.42 +/− 0.57	There were no significant differences between GAD and HC on overall task accuracy
Demenescu et al., 2013	Emotion recognition task	SAD (comorbidity with other psychiatric disorders) = 17 Comparison group HC = 16 Panic Disorder = 14 SAD + panic disorder = 8	33.07 +/−10.27 65%	35.56 +/−9.62 69%	SAD =12.88+/−3.52 HC = 13.44+/−2.87	Not reported	Not reported	There were no significant differences between SAD and HC on overall task accuracy.
Sladky et al., 2012	Emotion Discrimination task	SAD = 15 Comparison group: HC = 15	26.6 +/−8.6 47%	25.4 +/−3.4 53%	Not reported	Not reported	LSAS: SAD = 75.6 +/−22.7 HC = 5.3+/−7.3	There were no significant differences between SAD and HC on the overall task accuracy.
Montange et al., 2006	Emotion recognition task	SAD = 24 Comparison group: HC = 26	36.7 +/−10.4 58%	37.6 +/−12.7 54%	SAD = 16.8+/−0.8 HC = 17.2+/−2.7	BDI: SAD = 7.0+/−6.5 HC = 2.63+/−2.5	LSAS: SAD = 69.7 +/−15.6 HC = 13.1+/−10.4	There were no significant differences between patients and HC on the overall task accuracy.
Lundh et al., 1996	Facial memory task	SAD = 20 Comparison group: HC = 20	31.9 +/−8.9 80%	32.6 +/−10.6 80%	Range: 11–15	Not reported	ADIS: SAD = 7.05 +/− 1.19 HC = Not reported	There were no significant differences between SAD and HC on the recognition of faces during the face encoding task.

ERT: Emotion Recognition Task; FID: Facial emotion Identification; FER task: Ekman and Friesen photograph task; FEEST: The Facial Expressions of Emotions: Stimuli and Tests; DANVA-2-TW: The Diagnostic Analysis of Non-verbal Accuracy 2-Taiwan version; SAD: Social anxiety disorder; HC: Healthy controls; ADHD: *Attention deficit hyperactivity disorder*; MDD: Major depressive disorder; PTSD: Posttraumatic stress disorder; OCD: Obsessive–compulsive disorder; GAD: Generalized anxiety disorder; HAM-D: Hamilton Depression Rating Scale; DASS-21: Depression, Anxiety and Stress Scales; QIDS: Quick Inventory of Depressive Symptomatology; BDI: Beck’s Depression Inventory; LSAS: The Liebowitz Social Anxiety Scale; SIAS: Social interaction anxiety scale; PSWQ: Penn State Worry Questionnaire; ADIS: Anxiety disorder Interview Schedule.

**Table 2:** Characteristics of the studies included for TOM tests.

Author	TOM test	Principal diagnosis (*n*) and comparison groups (*n*)	Demographic characteristics			Clinical scales		Main results
			GAD/SAD mean age +/− SD Sex (female %)	HC mean age +/− SD Sex (female %)	Years of education mean +/− SD (GAD-SAD) (HC)	Depression scale mean +/− SD (GAD-SAD) (HC)	Anxiety scale mean +/− SD (GAD-SAD) (HC)	
Tetik et al., 2022	RMET	SAD (comorbidity with other psychiatric disorders) = 26 Comparison group: HC =26	27.68 +/−8.33 31%	27.15 +/− 6.69 23%	SAD = 15.15 +/− 2.92 HC =15.83+/− 2.64	Not reported	LSAS: SAD = 68.8 +/−20.35 HC = 23.7 +/−12	Mean total scores in the RMET were significantly lower in patients than HC
Küçükparlak et al., 2021	RMET	SAD = 47 Comparison group: HC = 50	Range: 18–60 Mean and SD not reported 40%	Range: 18–60 Mean and SD not reported 48%	SAD = 11.12 +/−2.81 HC =9.56 +/−0.62	BDI: SAD =16.28 ± 11.52 HC =6.76 ± 5.34	LSAS: SAD =55.485 +/−11 HC =36.45 +/−6.63	Mean total scores in the RMET were significantly lower in patients than HC
Maleki et al., 2020	RMET FPT	SAD (non-comorbid) = 35 Comparison groups: SAD comorbid MDD = 37 HC = 35	27.49 +/−2.06 46%	28.38 +/−3.41 49%	SAD = 14.36+/−1.70 HC = 16.21+/− 2.09	BDI: SAD = 20.36 +/−5.11 HC = 8.11 +/−3.28	BAI: SAD = 41.59 ± 6.13 HC = 7.62 ± 3.39	Both SAD and MDD patients performed lower than controls in the RMET test. No differences between SAD and HC groups were found in the FPT
Hendriks et al., 2020	FPT	SAD (comorbidity with other psychiatric disorders) = 39 Comparison group: HC = 39	34.1 SD not reported 62%	21.6 SD not reported 54%	Not reported	Not reported	Not reported	On the Faux-pas test, the clinical group showed superiority in accuracy over the control group
Zainal & Newman, 2019	RMET MASC	GAD (comorbidities in all participants) = 69 Comparison group: HC = 102	18.85 +/− 1.11 91%	19.04 + − 1.19 74%	Not reported	BDI: SAD = 22.25 +/−3.52 HC =34.10 +/−9.68	PSWQ: SAD =75.32 +/−11.5 HC = 44.89 +/−12.9	There were no significant differences between groups in the RMET or the MASC
Aydin et al., 2019	RMET	GAD (comorbid MDD) = 37 Comparison groups: Panic disorder = 44 HC = 50	36.35 +/− 11.37 70%	33.20 +/− 9.50 48%	GAD = 11.59 +/−4.46 HC = 12.20 +/− 3.95	Not reported	MCQ-30: GAD =76.97 +/−12 HC =70.29 +/−14.3	Patients with GAD significantly lower scores than HC in the RMET
Pepper et al., 2018	RMET FPT	SAD = 64 Comparison groups: Autism = 53 Early psychosis = 51 HC = 31	21.75 +/−4.38 47%	24.77 +/−6.08 39%	Not reported	DASS-21: SAD = 23.38 +/−11.22 HC =4.71+/−5.36	Not reported	There were no differences between patients with SAD and HC in any measure
Washburn et al., 2016	RMET MASC	SAD = 12 Comparison groups: SAD (comorbid MDD) =24 MDD = 40 HC = 43	SAD = 19.83 +/−4.11 58% SAD (comorbid MDD) = 19.71 +/−2.81 75%	18.74 +/−1.71 65%	Not reported	BDI-II: SAD = 16.92 +/−11.41 SAD (comorbid) = 19.71 +/− 11.19 HC = 7.35 +/−6.88	SAASA: SAD = 113.13 +/−20.12 SAD (comorbid) = 111.92 +/−19.91 HC = 77.12 +/−18.49	The non-comorbid SAD group was significantly less accurate at decoding mental states in the RMET than the MDD and HC. There were no significant differences between groups in the MASC task accuracy.
Buhlman et al., 2015	MASC	SAD (comorbidity with other psychiatric disorders) = 35 Comparison groups: Body dysmorphic disorder = 35 Obsessive compulsive disorder = 35 HC = 35	32.20 +/−8.85 60%	32.74 +/−10.98 49%	SAD = 16.14 +/−2.46 HC = 16.66 +/−1.85	BDI-II: Not reported	LSAS: SAD = 76.50 +/−23.40 HC = 25.88 +/−15.18	Participants with SAD showed significantly lower scores in the MASC than participants with body dysmorphic disorder, participants with obsessive–compulsive disorder, and HC.
Hezel & McNally 2014	RMET MASC	SAD (comorbid MDD) = 40 Comparison group: HC = 40	26.5 +/− 11.9 68%	20.1 +/−2.2 85%	Not reported	CESD: SAD = 20.70+/−13.8 HC = 6.35+/−6.63	LSAS: SAD = 72.48+/−22.35 HC =26.03+/−16.37	Participants with SAD had significantly lower scores in the RMET task. There were no significant differences in the MASC task accuracy

RMET, Reading the Mind in the Eyes; FPT, Faux Pas test; MASC, Movie Assessment of Social Cognition; SAD: Social anxiety disorder; HC: Healthy controls; MDD = major depression disorder; GAD: Generalized anxiety disorder; BDI: Beck’s Depression Inventory; DASS-21: Depression, Anxiety and Stress Scales; CESD: Center for Epidemiologic Studies Depression Scale; LSAS: The Liebowitz Social Anxiety Scale; BAI: Beck’s Anxiety Index; PSWQ: Penn State Worry Questionnaire; MCQ-30: Metacognition Questionnaire 30; SAASA: The Social Anxiety and Avoidance Scale for Adolescents.

### 2.4. Statistical analyses

Meta-analyses were conducted using OpenMeta [Analyst] software (32). First, we conducted meta-analyses (one for each domain) between the anxiety disorder groups (for both SAD and GAD patients) and healthy controls (HC) using random-effects models (DerSimonian-Lard estimate; significance at *p* < 0.05). The effect estimate was adjusted to standardized mean differences, depending on the magnitude of variation across studies (33). Heterogeneity was analyzed using the *I*^2^ statistics and the Cochrane’s Q (34). The *I*^2^ metric is independent of the number of studies and can be compared across meta-analyses with different numbers of studies and metrics (34, 35). For *I*^2^, a low heterogeneity corresponds to values between 0% and 25%; medium between 25% and 50%, and values greater than 50% indicate considerable heterogeneity (36). Cochrane’s *Q* is a non-parametric test that verifies if the differences between patients and controls are consistent for all evaluated studies. Because Cochrane’s *Q* is sensitive to the number of studies included, it may be underpowered for samples <20 (34, 37).

Second, we performed additional analyses for both domains excluding studies reporting GAD samples to determine their effect on the overall results on emotion recognition and ToM performances. It is important to note that some studies used more than one test of ToM, fulfilling inclusion criteria. We only included one result in the analyses to prevent reporting inconsistencies.

Third, for ToM analyses, given that the Reading the Mind in the Eyes (RMET) and the Movie Assessment of Social Cognition (MASC) were the most used measures across studies, we compared the performance of GAD and SAD patients vs. healthy controls in these tasks. Finally, we repeated these analyses for SAD patients vs. healthy controls.

#### 2.4.1. Meta-regression analyses

We conducted meta-regression analyses (38) to explore the relationship between relevant demographic and clinical variables with the primary emotion recognition and ToM outcomes. We included the following covariates: sex (represented as the percentage of females), the average age, the average years of education, and the rescaled anxiety scores. Since outcome measures varied in anxiety scales across studies, to aid interpretability, we rescaled all outcomes on a 0 to 100 scale, with the minimum scores represented as 0 and the highest as 100. This rescaling did not change the results; it was purely to allow for greater interpretability. Indeed, rescaling clinical outcomes on a 0 to 100 scale to aid interpretability is a standard procedure previously used in meta-analyses (39, 40) and clinical studies using scales with different ranges (41, 42).

### 2.5. Risk of bias assessment

To assess the risk of bias of the studies included in the meta-analysis, we used the Newcastle–Ottawa Scale (NOS) (43). The NOS evaluates the quality of non-randomized observational studies and has been utilized in several published systematic reviews and meta-analyses (44). This scale implemented a star system in which each study can receive up to a maximum of nine stars if all criteria have been satisfied in three categories: selection, comparability, and exposure or outcome. Higher scores indicate better quality. We used a cut-off value of ≥7 to define low risk of bias (45, 46).

### 2.6. Publication bias

We used funnel plots (47) and Egger’s test (48) to assess publication biases.

## 3. Results

### 3.1. Study selection

The initial search of databases for GAD yielded 97 studies. After the removal of duplicates (*n* = 44), 53 studies were screened. We excluded 45 studies, and eight met all inclusion criteria. Four of them were included in the systematic review and meta-analysis, the remaining were excluded because they did not report complete statistical data, and this was not provided by the corresponding authors. The PRISMA flow diagram in Figure 1 provides an overview of the study selection process for GAD.

For SAD, the initial search of databases yielded 1,030 studies. After the removal of duplicates (*n* = 729), 302 studies were screened. We excluded 274 studies, and 26 met all inclusion criteria. Twenty-one of them were included in the systematic review and meta-analysis, the remaining were excluded due to incomplete statistical data (*n* = 3) or lack of an ordinal measure of emotion recognition or ToM (*n* = 5). Incomplete statistical data was not provided by the corresponding authors. The PRISMA flow diagram in Figure 2 provides an overview of the study selection process for SAD.

Finally, 21 studies (GAD = 4 and SAD = 17) were included in this meta-analysis.

### 3.2. Study characteristics

The characteristics of included studies are shown in Tables 1, 2. As can be seen from these tables, 11 studies measured emotion recognition, 10 assessed ToM, and 2 included measures of both domains. For both domains, emotion recognition and ToM, most studies assessed patients with SAD. The final sample consisted of 178 patients diagnosed with GAD (mean age = 32.3 years, SD = 9.28, 79.2% female, education level = 14.4 years, SD = 2.7), 585 with SAD (mean age = 28.3 years, SD = 7.2, 51.1% female, education level = 14.3 years, SD = 2.5), and 753 healthy controls (mean age = 27.7 years, SD = 6.1, 57.5% female, education level = 14.5 years, SD = 2.0).

It is worth noting that, for the studies assessing emotion recognition in SAD patients, almost half of them (45.5%) included individuals with comorbid psychiatric disorders. The most common comorbidities were major depressive disorder, attention deficit hyperactivity disorder, panic disorder, and obsessive–compulsive disorder. The two studies assessing emotion recognition abilities in GAD did not include patients with comorbid disorders.

For studies assessing ToM in GAD or SAD, most of them (60%) included patients with comorbid psychiatric diagnoses. The most common comorbidity was major depressive disorder.

Regarding the emotion recognition and ToM outcome measures, it is relevant to highlight that different measurements were used by authors when examining the same construct (as shown in Tables 1, 2). On emotion recognition, all included studies assessed basic emotion recognition. Most of them included measures of recognition of static facial stimuli, fewer studies included dynamic stimuli such as video clips, and one of them included the assessment of emotions in prosody. On ToM, the reviewed studies used the Reading the Mind in the Eyes (RMET), the Movie Assessment of Social Cognition (MASC), or the Faux Pas Test (FPT). The most used ToM measure was the RMET (80% of the studies).

### 3.3. Meta-analytic results

#### 3.3.1. Emotion recognition

**Figure 3:**
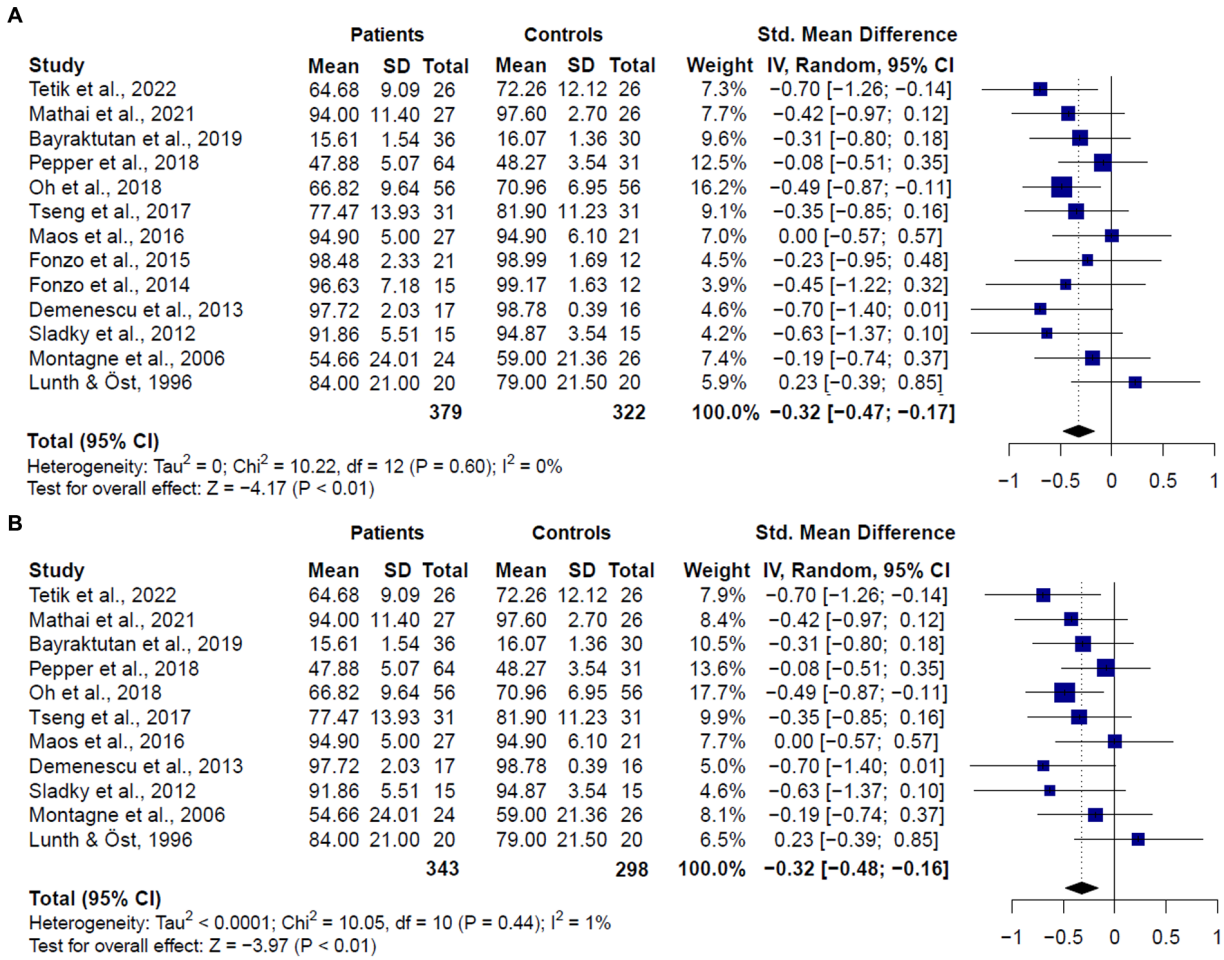
Forest plots showing effect size estimates for overall emotion recognition differences between **(A)** SAD and GAD patients and healthy controls **(B)** only SAD patients and healthy controls.

The meta-analysis including studies assessing emotion recognition in both patients diagnosed with GAD or SAD (*k* = 13) revealed that patients showed worse performance than healthy controls (SMD = −0.32, CI = −0.47 – 0.17, *z* = −4.17, *p* < 0.0001) (Figure 3A). No significant evidence for heterogeneity was found (*Q* = 10.22, df = 13, *p* = 0.60, *I*^2^ = 0%). It is worth mentioning that, despite the significant results, only two studies comparing SAD patients and healthy controls (13, 20) reported significantly worse performance in patients’ overall scores. Considering that the study by Oh et al. (13) was the one with the largest sample of patients and controls, and the higher weight in the results, we conducted a sensitivity analysis with the leave-one-out method. Results of this analysis showed that no single study was responsible for the pooled result of the meta-analysis (See Supplementary Figure 1).

When we compared only patients with SAD and healthy controls (*k* = 11), we also found significant differences between groups (SMD = −0.32, CI = −0.47 – −0.16, *z* = −3.97, *p* < 0.0001) (Figure 3B), and no significant evidence for heterogeneity (*Q* = 10.05, df = 10, *p* = 0.43, *I*^2^ = 1%). We did not compare patients with GAD and healthy controls since only two studies met inclusion criteria. These two studies (16, 17) did not report significant differences between patients and controls in overall emotion recognition performance.

#### 3.3.2. ToM

**Figure 4:**
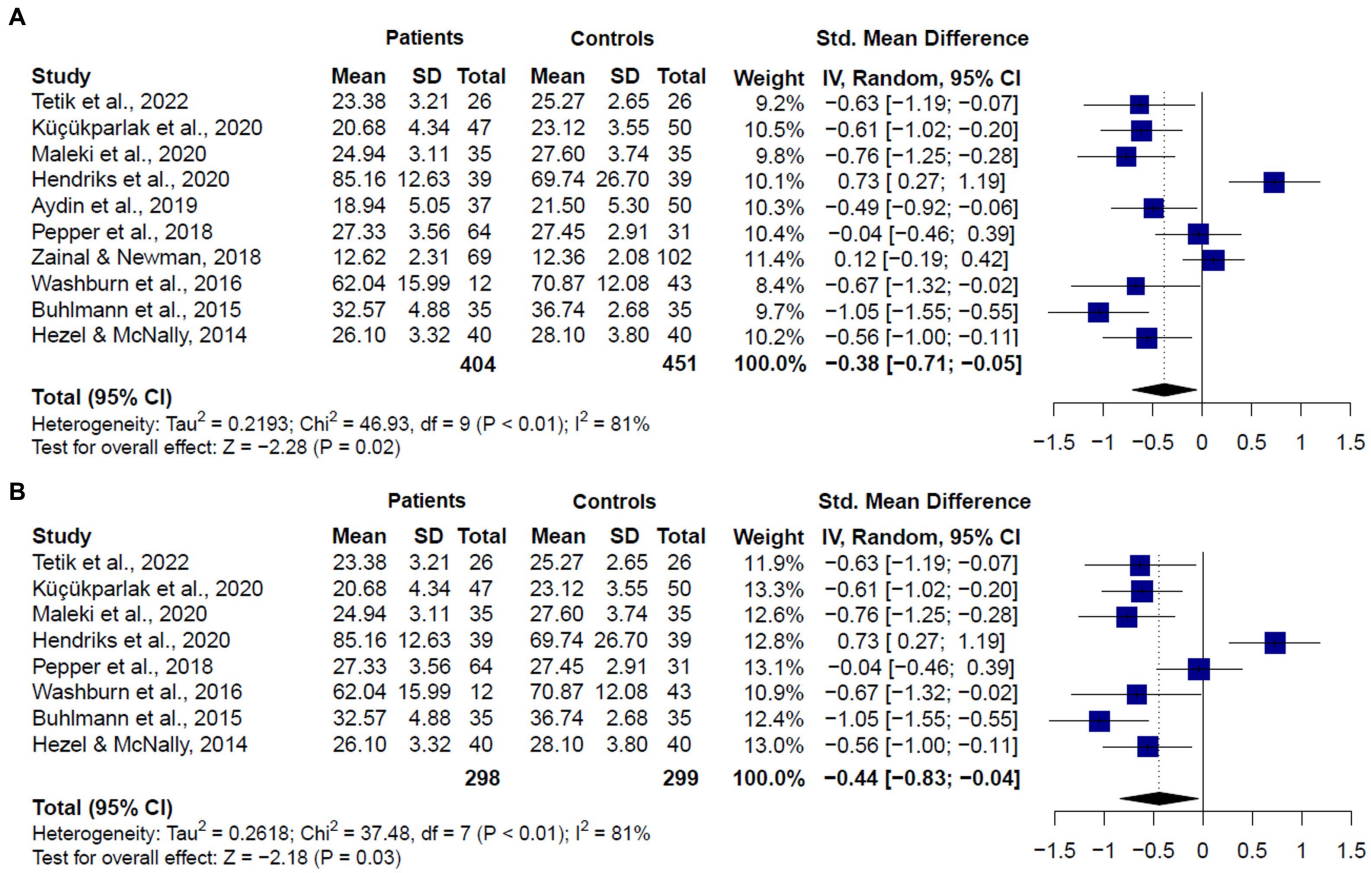
Forest plots showing effect size estimates for overall theory of mind differences between **(A)** SAD and GAD patients and healthy controls **(B)** only SAD patients and healthy controls.

The meta-analysis including studies assessing ToM in patients diagnosed with GAD or SAD together (*k* = 10) showed significant differences between groups, with patients showing lower scores than healthy controls (SMD = −0.38, CI = −0.71 – 0.005, z = −2.280, *p* < 0.01) (Figure 4A). Significant and substantial heterogeneity was observed (*Q* = 46.12, df = 9, *p* < 0.001, *I*^2^ = 81%). It is worth noting that only two studies (6, 26) showed no significant differences between GAD or SAD patients and healthy controls in ToM abilities using the RMET. Seven studies (20, 22–25, 28, 49) reported significantly lower performance in patients, three of them used the RMET, three used the RMET and the MASC, and one, the RMET and the FPT. Only one study, using the FPT, found that SAD patients exhibited higher accuracy than the control group.

When we compared only patients with SAD and healthy controls (*k* = 8), we also found significant differences between groups (SMD = −0.44, CI = −0.83 – 0.04, *z* = −2.18, *p* < 0.01) (Figure 4B), and substantial heterogeneity (*Q* = 38.27, df = 7, *p* < 0.01, *I*^2^ = 81.7%). We did not compare patients with GAD and healthy controls, given only two studies met inclusion criteria. These studies showed mixed results. One of them (26) found no significant differences between GAD patients and healthy controls in the RMET or the MASC overall performances. The other one (22) reported significant lower performance in GAD patients in the RMET total score.

**Figure 5:**
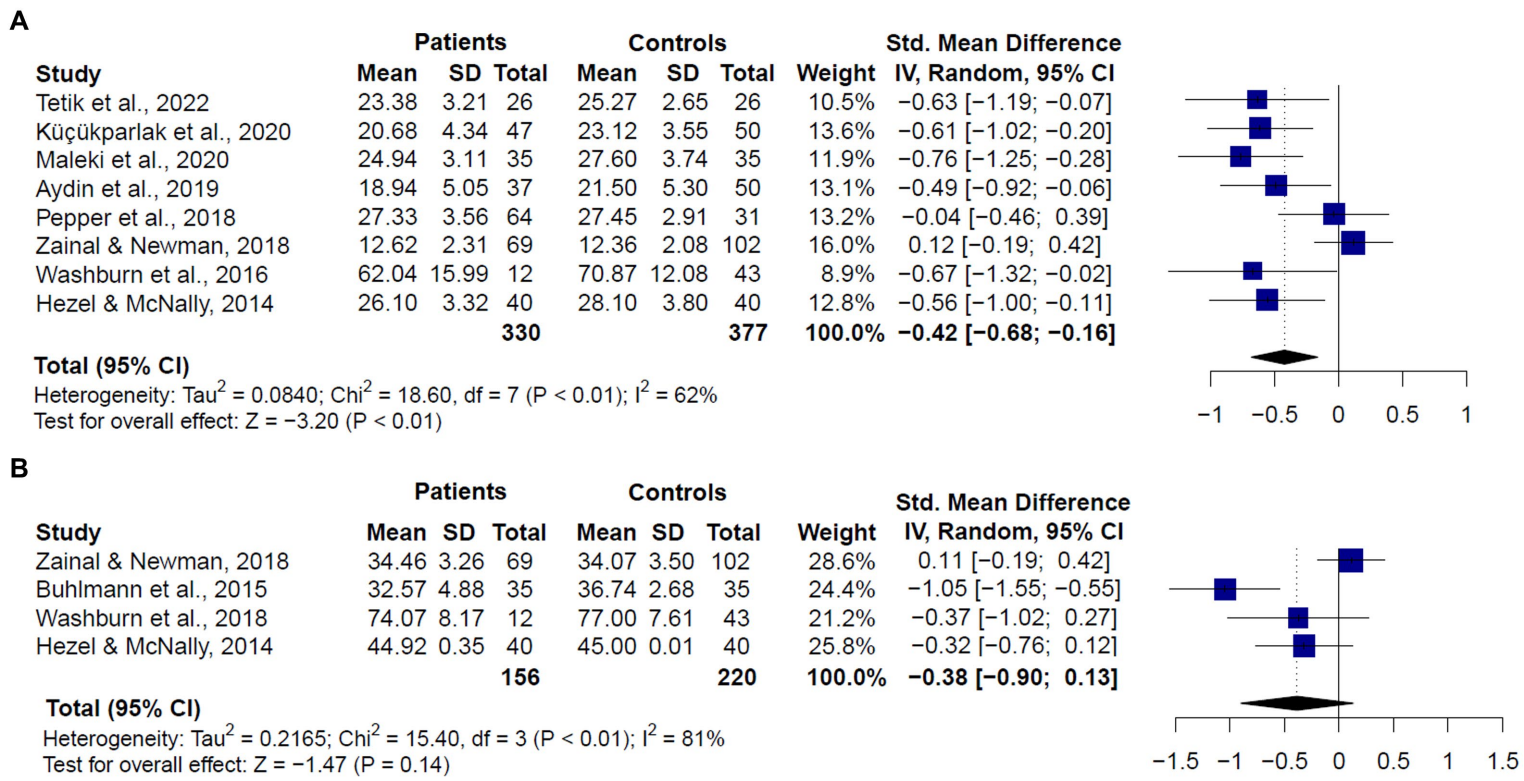
Forest plots showing effect size estimates for overall theory of mind differences between SAD and GAD patients and healthy controls in **(A)** the Reading the Mind in the Eyes (RMET), and **(B)** the Movie Assessment of Social Cognition (MASC).

Given that the RMET and the MASC were the most used measures of ToM across included studies, we also compared the performance of GAD and SAD patients vs. healthy controls in these tasks (Figure 5). For the RMET (*k* = 8), results revealed that patients performed significantly worse than controls (SMD = −0.426, CI = −0.68 – −0.16, z = −3.20, *p* < 0.01). We still found significant heterogeneity, but less than the one observed when comparing studies with different ToM tasks (*Q* = 18.52, df = 7, *p* < 0.01, *I*^2^ = 62.2%) (Figure 5A). For the MASC (*k* = 4), we did not find significant differences between patients and controls (SMD = −0.38, CI = −0.90 –0.13, *z* = −1.47, *p* = 0.14), and there was significant and substantial heterogeneity (*Q* = 15.40, df = 3, *p* < 0.01, *I*^2^ = 81%) (Figure 5B).

**Figure 6:**
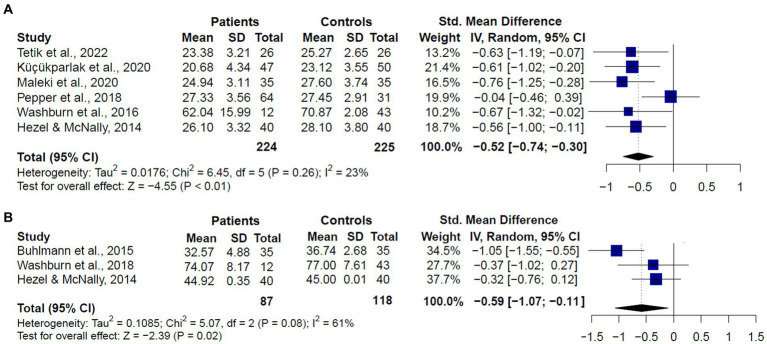
Forest plots showing effect size estimates for overall theory of mind differences between only SAD patients and healthy controls in **(A)** the Reading the Mind in the Eyes (RMET), and **(B)** the Movie Assessment of Social Cognition (MASC).

When we analyzed the performance of SAD patients compared to healthy controls (*k* = 6), we also found that patients performed significantly worse in the RMET (SMD = −0.52, CI = −0.74 – −0.30, *z* = −4.55, *p* < 0.01), and no significant evidence for heterogeneity was found (*Q* = 8.97, df = 5, *p* = 0.26, *I*^2^ = 23.2%) (Figure 6A). For the MASC (*k* = 3), we also found significantly worse performance in SAD patients than controls (SMD = −0.59, CI = −1.07 – −0.11, z = −2.39, *p* = 0.02). However, considerable heterogeneity was found (*Q* = 5.07, df = 2, *p* = 0.08, *I*^2^ = 61%) (Figure 6B).

We did not compare the performance of patients with GAD and healthy controls in the RMET and the MASC due to the low number of studies meeting inclusion criteria.

### 3.4. Meta-regression analyses

#### 3.4.1. Emotion recognition

We conducted meta-regression analyses to explore the relationship between relevant variables and emotion recognition performance in GAD and SAD patients. Results showed that emotion recognition abilities were not significantly influenced by age (*k* = 13, *z* = 0.58, *p* = 0.55, *R*^2^ = 0.00), sex (*k* = 13, *z* = 0.35, *p* = 0.72, *R*^2^ = 0.00), years of education (*k* = 9, *z* = −1.09, *p* = 0.27, *R*^2^ = 0.00), or severity of anxiety (*k* = 10, *z* = −0.06, *p* = 0.94, *R*^2^ = 0.00).

#### 3.4.2. ToM

We also performed meta-regression analyses to explore the relationship of relevant variables with ToM performance in GAD and SAD patients. Results showed that ToM performance was not significantly dependent on age (*k* = 10, *z* = −0.65, *p* = 0.51, *R*^2^ = 0.00), sex (*k* = 10, *z* = 1.04, *p* = 0.29, *R*^2^ = 1.36), years of education (*k* = 5, *z* = −1.15, *p* = 0.24, *R*^2^ = 0.00), or severity of anxiety (*k* = 8, *z* = 0.53, *p* = 0.59, *R*^2^ = 0.00).

### 3.5. Risk of bias assessment

The quality assessment results are shown in Supplementary Table 1. The mean score was 7.80 (SD = 0.82), 20 of the 21 included studies were awarded ≥7 stars and considered to be of low risk of bias.

### 3.6. Publication bias

We used funnel plots and Egger’s test to assess publication bias. For emotion recognition, funnel plot was symmetric and Egger’s test non-significant (intercept = −0.17; *p* = 0.69), suggesting no publication bias for this set of studies (see Supplementary Figure 2). For ToM, visual inspection of the funnel plot showed some asymmetry suggesting the presence of small studies effect (see Supplementary Figure 3). However, the Egger’s test was not significant (intercept = 0.75, *p* = 0.16), indicating insufficient evidence for publication bias.

## 4. Discussion

This is the first meta-analysis of emotion recognition and ToM abilities in adults with anxiety disorders, including both GAD and SAD. Results of the 21 studies included in this meta-analysis showed that compared to healthy controls, patients with SAD exhibited impairments in emotion recognition and ToM. Results for GAD were not conclusive and should be interpreted with caution, given that these social cognition domains have been less studied in these patients, with only four studies fulfilling the inclusion criteria (two for each domain). In addition, meta-regression analyses indicated that relevant demographic and clinical variables (age, sex, education level, and anxiety scores) were not associated with emotion recognition or ToM impairments of GAD and SAD patients.

### 4.1. Emotion recognition

The meta-analysis of studies assessing emotion recognition in patients diagnosed with GAD or SAD showed that patients exhibited significantly worse performance than healthy controls. It is worth mentioning that, despite the significance of this meta-analysis, only two studies comparing SAD patients and healthy controls (13, 20) reported significantly worse performance in patients’ overall scores. Considering that the study from Oh et al. (13) was the one with the largest sample of patients, and the highest weight in the results, we conducted a sensitivity analysis with the leave-one-out method. The results were very similar to the original ones, indicating that the study by Oh et al. (13) neither other single study was responsible for the pooled result of this meta-analysis. Thus, although the study by Oh et al. (13) was the one with the most precise estimates, the significant effects shown in the current meta-analysis seem to be guided by the fact that all studies, except for the one by Lundh and Öst (18), showed a lower mean performance in patients compared to controls. We also conducted the analysis for studies assessing patients with SAD only, and the results remained similar to the original ones.

It is striking to notice the diversity of tests used to assess the recognition of emotions in the included studies. Some studies used static visual facial stimuli; in some studies, the intensity of emotions varied; in other articles, the facial expressions showed the same emotion intensity; and some used moving images for the assessment. In addition, methods of presenting photographs varied concerning the total number of photographs, the time presented, and so on. Although these differences may have affected the results and data interpretation, these methodological differences did not seem to represent a source of heterogeneity.

It is noteworthy that, from the 13 included studies, only two assessed patients with GAD (16, 17). These two studies reported no significant differences between patients and healthy controls in overall emotion recognition scores, suggesting that this domain seems to be preserved in patients with GAD. However, it is essential to highlight that the same authors have conducted some studies including small sample sizes (15 and 21, respectively) of patients without psychiatric comorbidities and used a task in which participants were instructed to match the target facial expression to one of two faces. These factors could have an impact the results. Thus, research on emotion recognition abilities in patients with GAD is still lacking and should be further investigated in future studies with larger samples and more ecologically valid and demanding tasks.

Our results are not consistent with those of the only previous meta-analysis assessing emotion recognition impairments in adults with anxiety disorders (7). Some methodological factors may partially explain these differences. First, only one study (50) was included in both meta-analyses. Although we contacted the respective authors, we could not obtain the data for the remaining six studies analyzed by Plana et al. (7). Second, our work includes more recent evidence since only four of the 21 included studies were published before 2014. Thus, the samples analyzed here and in the previous meta-analysis notably differ, which probably explains the uneven results.

Although most of the studies included in the current meta-analysis did not provide the data to analyze positive and negative emotions recognition independently, it is remarkable that two studies did not found significant differences between SAD patients and healthy controls in overall scores (11, 14), but reported significant impairments in particular emotions. Specifically, Maoz et al. (2016) reported that SAD participants judged a significantly higher proportion of faces as angry, and Tseng et al. (14) found that SAD participants exhibited significantly lower accuracy in recognizing facial and prosodic emotions of fear. These results are consistent with those of a previous review (51) showing that facial emotion processing in SAD is influenced by a negative bias, that is, affected individuals tend to manifest peculiar processing of pictures with negative facial displays of emotion. Thus, the emotional valence of facial stimuli is one factor to be considered in SAD assessment. The lack of available data to further explore the influence of this factor, highlights the importance of addressing the recognition of particular emotions in future meta-analyses.

We also explored the relationship between emotion recognition in GAD and SAD patients and age, sex, years of education, and anxiety levels. Meta-regression results showed that emotion recognition impairments did not depend on any of these variables. As data regarding years of education was not reported by several studies, result on this meta-regression should be considered with caution. Similarly, data on anxiety levels was not included in all studies and there were differences regarding the instruments used to assess this variable. However, one of the reviewed studies (52) reported a significant association between symptom severity of social anxiety and functional connectivity between brain regions involved in perception of fearful faces (amygdala and medial prefrontal cortex). Thus, the potential relationship between anxiety severity and emotion recognition impairment should be further investigated in patients with GAD and SAD.

### 4.2. Theory of mind

This is the first meta-analysis addressing ToM in adults diagnosed with GAD or SAD. The analysis comparing both groups, GAD and SAD, and healthy controls revealed that patients showed significantly lower performance in ToM tasks. These results were similar when we analyzed only the studies assessing SAD patients. However, in both cases, significant and substantial heterogeneity was observed.

Considering that the diversity of tests used to assess ToM may be one of the possible sources of heterogeneity, we conducted the analyses independently for studies that used the RMET or the MASC. When we compared both groups of patients and controls, we found that patients performed significantly worse than controls in the RMET, but there were no significant differences in the MASC total scores. For both analyses, we still found significant heterogeneity, but less than the one observed when comparing studies using different ToM tasks. This suggests significant heterogeneity seems to be associated with both the diversity of ToM tasks and the differences between SAD and GAD samples. Interestingly, when we analyzed only the SAD group compared to healthy controls, we found that patients showed significantly lower performance in both tasks, the RMET and the MASC. For studies using the RMET, there was low and non-significant heterogeneity. However, for studies using the MASC, heterogeneity was considerable. These differences in heterogeneity in the results of studies using RMET and MASC may be explained by the low number of studies employing the second measure (k = 3) and the heterogeneity in the samples assessed in these three studies. Two of them included patients with SAD and comorbid psychiatric conditions (53, 54) and the other one (55) assessed patients with SAD without comorbidities but with a very small the sample size (*n* = 12). Considering these limitations of previous studies, further investigation is needed on ToM abilities assessing more representative and homogenous samples of SAD patients.

Overall, our results suggest that ToM impairments are present in patients with SAD, regardless of the tasks employed in the assessment of this domain (RMET or MASC). Thus, ToM deficits in SAD seem to include more basic decoding abilities to detect emotion, such as the ones measured by the RMET (e.g., visual emotion detection using only the eyes), and the more complex affective and cognitive abilities to understand others’ emotions, intentions, and behaviors from multiple channels of data (i.e., video unfolding with auditory, visual, and interactive facial and body movement), such as the ones assessed by the MASC. Given that most of the studies included in the current meta-analysis did not provide the data to analyze affective and cognitive ToM abilities independently, differences in these domains should be addressed in future studies.

For GAD patients, results are not conclusive. One study using the RMET showed impairments (22), and the other one, using the RMET and the MASC, failed to find significant differences between patients and healthy controls in any task (26). Further studies are needed to determine whether the ToM abilities are impaired in patients with GAD.

Meta-regression results showed that age, years of education, or anxiety levels were not significantly correlated with ToM performance in GAD and SAD patients. However, the influence of education and anxiety severity to ToM should be considered with caution, given that these data were not provided by all included studies. In particular, the specific relationship between symptom severity and performance in the RMET was assessed by one of the included studies showing no significant associations in patients with GAD (56). Further studies are needed to understand the influence of demographic and severity of symptoms on ToM abilities in patents with SAD and GAD.

### 4.3. Limitations

Some of the most significant methodological limitations of research in this area are the limited number of studies and the small sample sizes available for entry into the meta-analysis, especially for GAD. Also, many of the studies fulfilling the inclusion criteria did not report complete statistical data. While efforts were made, these studies were not included despite multiple attempts to contact their authors. There was also substantial variability in measurement tools used to assess emotion recognition and ToM. In particular, for emotion recognition, different studies adopted different instruments to assess the same construct. Although all of them assessed basic emotions recognition and the majority used facial stimuli, the type of stimuli and the methods of presenting stimuli varied among them. These differences may have affected the current results.

In addition, one factor that might influence the current results is the frequent presence of comorbidities between the different disorders and in the same patient. Almost half of the studies’ samples included in this meta-analysis comprised patients with comorbid psychiatric disorders (13–15, 20, 22–24, 26, 52). Although lifetime comorbidity in patients with anxiety disorders occurs in more than 80% (57), variations in the composition of the samples across studies result in a relevant disadvantage for between studies comparisons, highlighting the need for future research assessing social cognition in more homogeneous samples GAD and SAD patients. Although it is expected that the presence of comorbid disorders would be associated with deeper impairments in emotion recognition and ToM, this has not been studied in GAD or SAD patients. Future research should further explore the impact of comorbid disorders on social cognition abilities of GAD and SAD patients.

Additionally, we investigated some variables (age, sex, education level, and severity of anxiety) that may affect emotion recognition or ToM performances (58–60). However, other factors (such as the presence of comorbidities, prior substance abuse, and executive functioning) were not examined. Given that most studies did not report data on these variables, future research should address the relationship between said factors and emotion recognition and ToM abilities of patients with SAD and GAD. Finally, only studies published in English were included in this review, which may result in limited generalizability of results.

### 4.4. Implications and future directions

Our meta-analytic findings revealed that patients with SAD exhibited impairments in basic emotions recognition and ToM. Accordingly, individuals with SAD are unable to identify basic emotions or make inferences about the thoughts of others, thereby misunderstanding social situations in everyday life. Consistently, research on cognitive biases suggests that individuals with SAD may lack an accurate view of how they are perceived by others, especially in social situations when they allocate attentional resources to monitoring their own actions as well as external threat (24). In addition, it has been suggested (14) that SAD patients take longer than healthy participants to recognize emotions across modalities which may imply a longer cognitive elaboration process and facilitate their avoidance responses. As the underlying mechanisms for emotion recognition and ToM impairments in SAD are unknown, further investigation is needed. Also, future meta-analytic studies should test whether patients with SAD are more likely to attribute more intense emotions and greater meaning to what others were thinking and feeling.

It is well established that impaired emotion recognition and ToM may contribute to significant everyday social difficulties, including reduced social competence, social isolation, and poorer social integration (61). In particular, this meta-analytic evidence suggests that emotion recognition and ToM may underlie interpersonal impairments observed in SAD patients. Despite this, it is important to recognize the limitations of employed social cognitive tests, including issues concerning ecological validity (62–64). Future studies should assess emotion recognition, ToM, and other social cognition domains in patients with SAD using more ecological tests. Also, our findings underscore the importance of routine clinical screening for social cognition in patients with SAD, as well as a critical need to develop evidence-based treatments for social cognitive impairment in this population.

Regarding GAD, results are not conclusive. Due to the small number of studies involving measures of emotion recognition and ToM in GAD patients, meta-analysis of these social cognitive domains was not possible. However, some previous studies (22, 65) and the only previous meta-analysis (7) found social cognition impairments in patients with GAD. These social cognition impairments may be associated with interpersonal problems in everyday life. These problems may be also linked with the excessive and uncontrollable worry which is the hallmark symptom of GAD (66). For example, people with GAD seem to be more likely than controls to either under- or over-estimate their impact and hostile behaviors on others (66). Considering the limited evidence, further research is urgently needed on social cognition in patients with GAD.

## 5. Conclusion

The findings of the current study synthesize the body of literature on emotion recognition and ToM in patients with SAD or GAD. Overall, our meta-analytic findings reveal that both of these domains are impaired in patients with SAD. Results for GAD are not conclusive due to the small number of studies involving measures of emotion recognition and ToM. Further studies employing ecological measures with larger and homogenous samples are needed to better delineate related factors influencing social cognition outcomes in patients with SAD and GAD. Such efforts will be beneficial for informing the design and implementation of evidence-based treatments for social cognitive impairment in these patients.

## Author contributions

SB, MT, and DF designed the study and supervised study conduct. GD and MAT performed the title and abstract review. SB, GD, and MAT reviewed the full texts and agreed on incusion/exclusion. GD and MAT contacted authors for missing data. MAT analyzed the data under supervision of SB and DF. SB drafted the manuscript. All authors contributed to the article and approved the submitted version.

## Funding

This work was supported by Ministerio de Ciencia, Tecnología e Innovación (Minciencias, Colombia) (grant # 915, 2019, and 123384466952).

## Conflict of interest

The authors declare that the research was conducted in the absence of any commercial or financial relationships that could be construed as a potential conflict of interest.

## Publisher’s note

All claims expressed in this article are solely those of the authors and do not necessarily represent those of their affiliated organizations, or those of the publisher, the editors and the reviewers. Any product that may be evaluated in this article, or claim that may be made by its manufacturer, is not guaranteed or endorsed by the publisher.
