# Supplementary material for: Performance in emotion recognition and theory of mind tasks in social anxiety and generalized anxiety disorders: a systematic review and meta-analysis
Source: Front Psychiatry. 2023 May 19;14:1192683. doi: 10.3389/fpsyt.2023.1192683 (PMC10235477; doi:10.3389/fpsyt.2023.1192683)
Supplement: Supplementary file 1 [file Data_Sheet_1.docx]

**Performance in emotion recognition and theory of mind tasks in social anxiety and generalized anxiety disorders: A systematic review and meta-analysis**

**Running title: social cognition in anxiety disorders**

**Sandra Baez^1*^, María Alejandra Tangarife^1^, Gabriela Dávila-Mejía^1^, Martha Trujillo-Güiza^2^, Diego A. Forero^3^,**

**^1^ Universidad de los Andes, Bogotá, Colombia**

**^2^ Facultad de Medicina, Universidad Antonio Nariño, Bogotá, Colombia**

**^3^ School of Health and Sport Sciences, Fundación Universitaria del Área Andina, Bogotá, Colombia**

***Correspondence:**

Sandra Baez, Ph.D., Universidad de los Andes, Carrera 1 # 18A-12, Bogotá, Colombia, Postal Code 111711.

Phone/Fax: +57 (1) 3394949 (ext.5560).

[sj.baez@uniandes.edu.co](mailto:sj.baez@uniandes.edu.co)


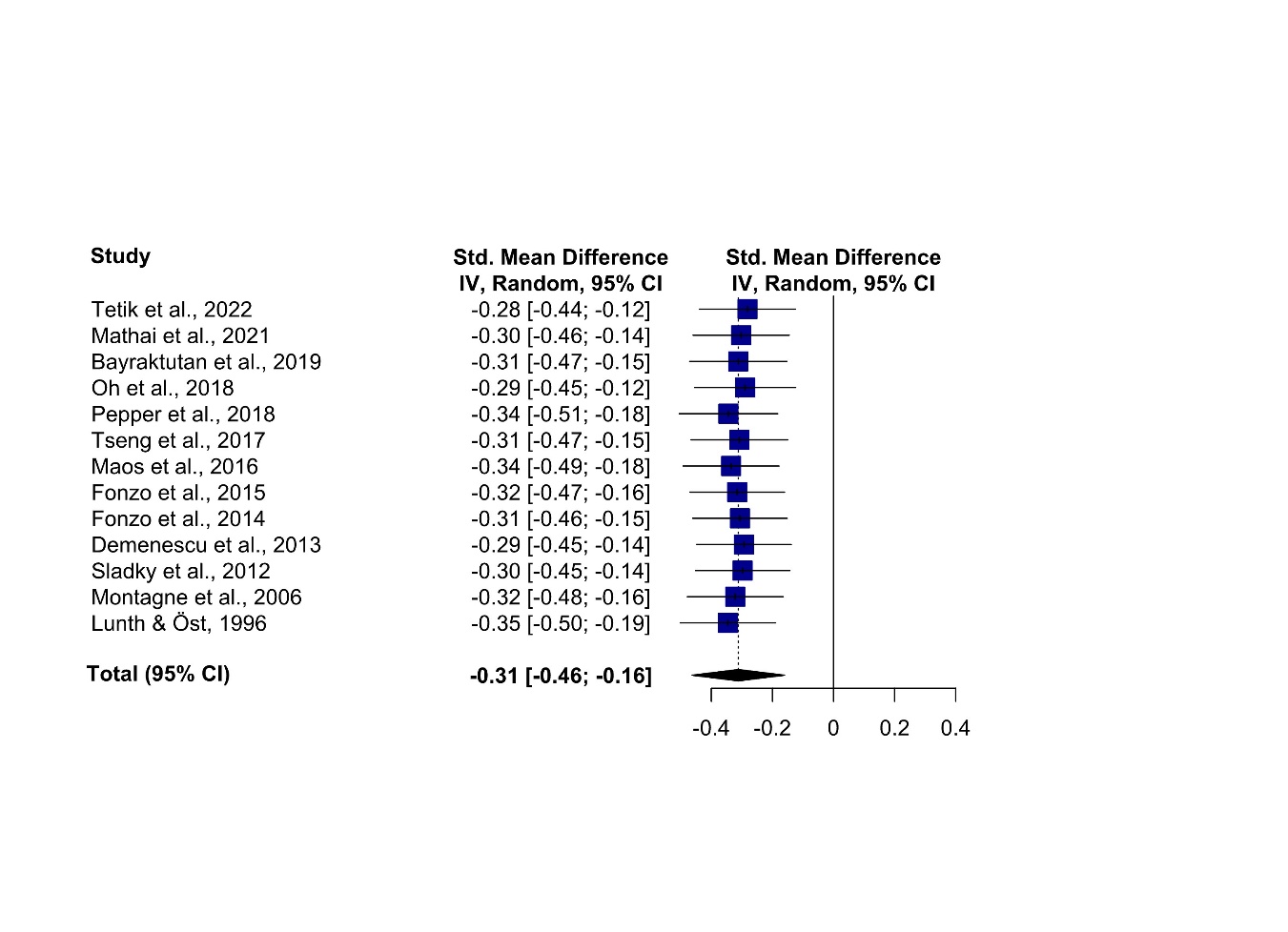


**Supplementary Figure 1.** Sensitivity analysis for the meta-analysis of emotion recognition in SAD and GAD patients


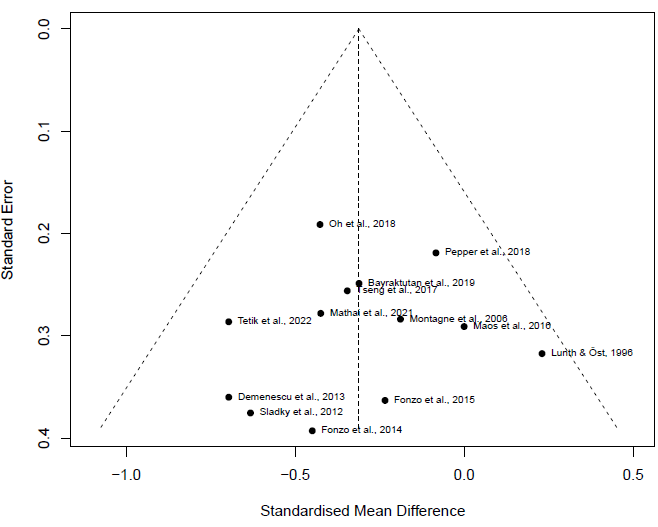


**Supplementary Figure 2.** Funnel plot for meta-analysis of emotion recognition in SAD and GAD.


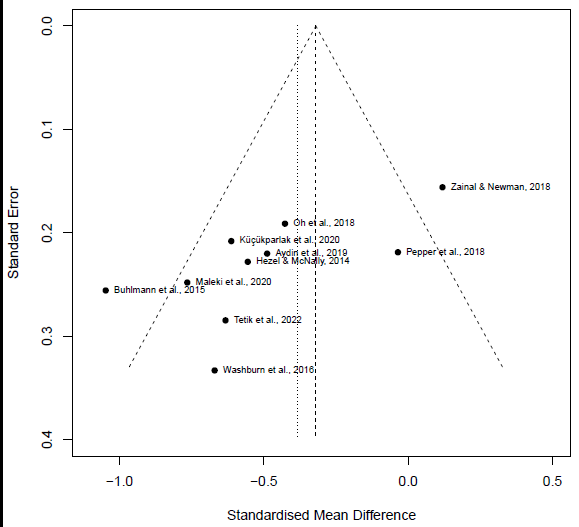


**Supplementary Figure 3.** Funnel plot for meta-analysis of emotion recognition in SAD and GAD

**Supplementary Table 1**. Newcastle–Ottawa Scale for risk of bias assessment
